# Supplementary figures and images for: Association between the Maternal Gut Microbiome and Macrosomia
Source: Biology (Basel). 2024 Jul 28;13(8):570. doi: 10.3390/biology13080570 (PMC11351347; doi:10.3390/biology13080570)

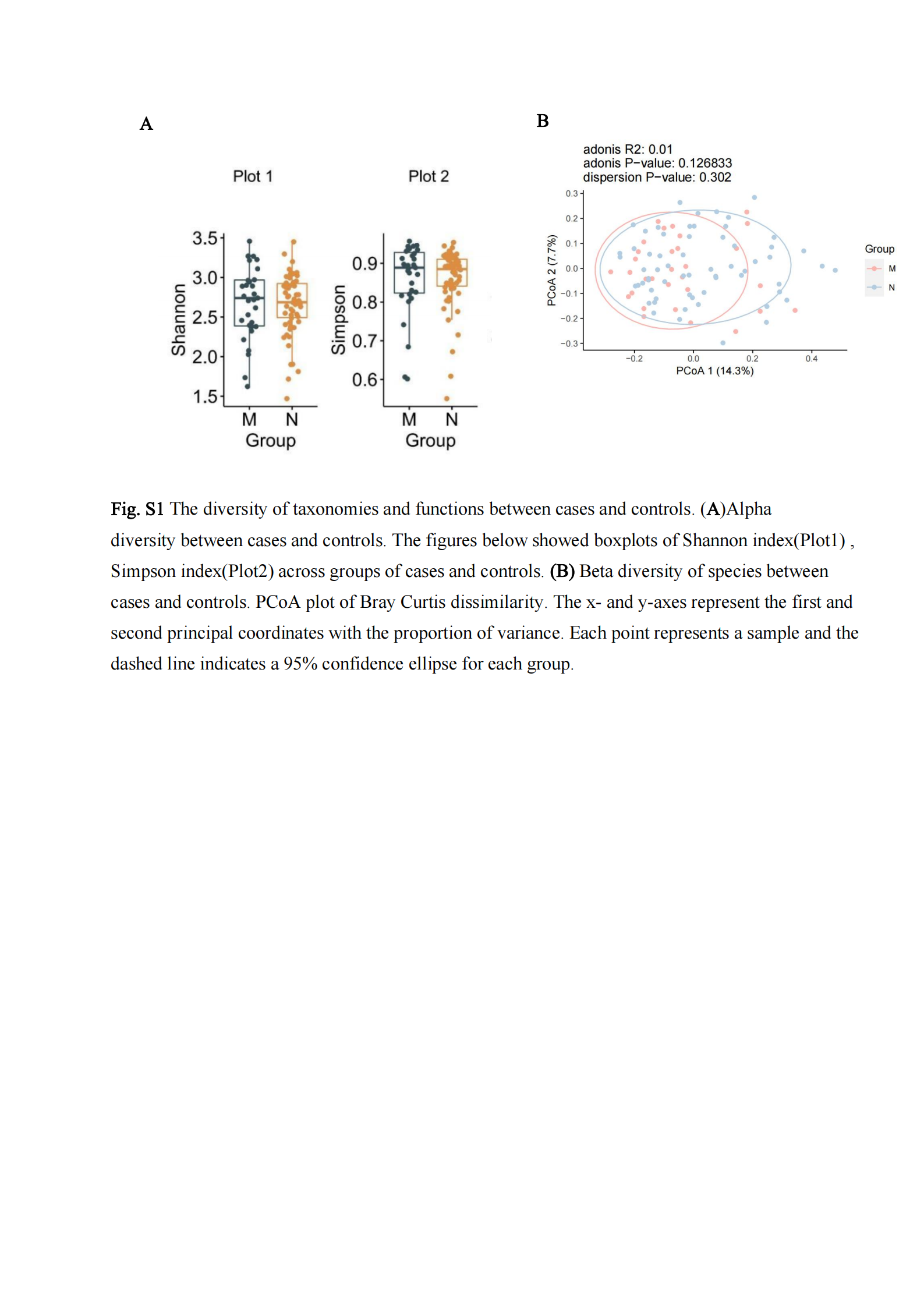

Supplement: Supplementary file 1 [file biology-13-00570-s001.zip › supplementary file/Additional Figure S1.tif]
